# Causes of fever in primary care in Southeast Asia and the performance of C-reactive protein in discriminating bacterial from viral pathogens

**DOI:** 10.1016/j.ijid.2020.05.016

**Published:** 2020-07

**Authors:** Thomas Althaus, Janjira Thaipadungpanit, Rachel C. Greer, Myo Maung Maung Swe, Sabine Dittrich, Pimnara Peerawaranun, Pieter W. Smit, Tri Wangrangsimakul, Stuart Blacksell, Jonas M. Winchell, Maureen H. Diaz, Nicholas P.J. Day, Frank Smithuis, Paul Turner, Yoel Lubell

**Affiliations:** a Mahidol-Oxford Tropical Medicine Research Unit (MORU), Faculty of Tropical Medicine, Mahidol University, Bangkok, Thailand; b Centre for Tropical Medicine and Global Health, Nuffield Department of Clinical Medicine, University of Oxford, Oxford, United Kingdom; c Myanmar-Oxford Clinical Research Unit (MOCRU), Medical Action Myanmar (MAM), Yangon, Myanmar; d Foundation for Innovative New Diagnostics (FIND), Geneva, Switzerland; e Maasstad Ziekenhuis Hospital, Department of Medical Microbiology, Rotterdam, The Netherlands; f Public Health Laboratory (GGD), Amsterdam, The Netherlands; g Division of Bacterial Diseases, Centers for Disease Control and Prevention, Atlanta, GA, USA; h Cambodia-Oxford Medical Research Unit (COMRU), Angkor Hospital for Children, Siem Reap, Cambodia

**Keywords:** C-reactive protein, Antibiotic prescription, Primary care, Southeast Asia, Causes of fever

## Abstract

**Objectives:**

This study investigated causes of fever in the primary levels of care in Southeast Asia, and evaluated whether C-reactive protein (CRP) could distinguish bacterial from viral pathogens.

**Methods:**

Blood and nasopharyngeal swab specimens were taken from children and adults with fever (>37.5 °C) or history of fever (<14 days) in Thailand and Myanmar.

**Results:**

Of 773 patients with at least one blood or nasopharyngeal swab specimen collected, 227 (29.4%) had a target organism detected. Influenza virus type A was detected in 85/227 cases (37.5%), followed by dengue virus (30 cases, 13.2%), respiratory syncytial virus (24 cases, 10.6%) and *Leptospira* spp. (nine cases, 4.0%). Clinical outcomes were similar between patients with a bacterial or a viral organism, regardless of antibiotic prescription. CRP was higher among patients with a bacterial organism compared with those with a viral organism (median 18 mg/L, interquartile range [10–49] *versus* 10 mg/L [≤8–22], *p* = 0.003), with an area under the curve of 0.65 (95% CI 0.55–0.75).

**Conclusions:**

Serious bacterial infections requiring antibiotics are an exception rather than the rule in the first line of care. CRP testing could assist in ruling out such cases in settings where diagnostic uncertainty is high and routine antibiotic prescription is common.

The original CRP randomised controlled trial was registered with ClinicalTrials.gov, number NCT02758821.

## Highlights

• Evidence on causes of fever is limited in Southeast Asia, especially in primary care.
• Point-of-care diagnostic tools could guide health workers’ antibiotic prescription.
• C-reactive protein was significantly increased in cases of bacterial infections.
• Most primary care patients recovered regardless of antibiotic prescription.
• Antibiotic prescription should be an exception in the primary levels of care.

## Introduction

Fever is a common reason for seeking health care in Southeast Asia. As malaria incidence declines, bacteria and viruses now represent the main contributors to acute febrile illness (World Health Organization, 2017, Chheng et al., 2013, Mayxay et al., 2015, Southeast Asia Infectious Disease Clinical Research Network, 2017, Wangdi et al., 2019). Identifying these pathogens is challenging, even in well-resourced laboratories with specialised staff, and most aetiological data for febrile illness originate in tertiary hospitals (Shrestha et al., 2018). Hospitalised patients are by definition more severely ill, often with important comorbidities, implying that findings may not be applicable to febrile patients attending primary levels of care.

Primary care in low-middle income countries is typically characterised by a shortage in human resources, diagnostics and evidence-based guidelines (Mendelson et al., 2016). Studies investigating causes of fever in this environment are few and frequently of poor quality: enrolment is often limited to a single clinical presentation and specific age category, and microbiological investigations rarely use gold-standard methods (Mueller et al., 2014, Kasper et al., 2012, Capeding et al., 2013). Additionally, most patients attend primary care early after symptom onset with non-severe presentations, lowering the chances of detecting a pathogen (Mueller et al., 2014, Kasper et al., 2012, Capeding et al., 2013, Schmidt et al., 2014, Cunnington, 2015, Waterer and Rello, 2011, Hackett et al., 2002, D’Acremont et al., 2014, Hildenwall et al., 2016). Empiric treatment guidelines are therefore based on limited epidemiological evidence and are often implemented by insufficient and poorly trained staff, contributing to irrational antibiotic prescription practices (WHO, 2006, Chen et al., 2004, Kanchanachitra et al., 2011).

High prescription levels are partly driven by frequent clinical overlap between bacterial and viral infections, challenging the identification of patients who might benefit from antibiotics (Chheng et al., 2013, Mayxay et al., 2015, Koh et al., 2010). Given these limitations in clinical judgment and laboratory structures, point-of-care testing (POCT) to guide fever management could be beneficial in primary care settings (WHO, 2011). Pathogen-specific tests represent one such option but several barriers undermine their potential use: these only exist for a small number of pathogens, with inconsistent performance, and most are antibody detection-based that might preclude the distinction between active infection and past exposure (Saraswati et al., 2018, Picardeau et al., 2014). A few antigen detection-based POCTs exist but their integration in low-level care is unrealistic: a *Salmonella typhi* rapid test requires laboratory infrastructure with poor detection in blood, even at high concentrations, and results are not available before 24–48 h (Castonguay-Vanier et al., 2013, Kuijpers et al., 2018); test sensitivities for influenza virus A, respiratory syncytial virus (RSV) and group A *Streptococcus* antigen-based POCTs are inconsistent (Drexler et al., 2009, Trombetta et al., 2018, Chartrand et al., 2015, Cohen et al., 2016); and accurate dengue antigen-based RDTs have not been found to be cost-effective in resource-poor settings (Lubell et al., 2016, Lim et al., 2017).

Non-specific host biomarkers measure the host-response to stimuli, and have been evaluated in the context of fever to discriminate between bacterial and viral pathogens (Kapasi et al., 2016). C-reactive protein (CRP) is one of the most studied host-response biomarkers of bacterial infection, consistently showing high sensitivity and moderate specificity, and CRP POCTs have been shown to be cost-effective in resource-poor environments (Lubell et al., 2016, Kapasi et al., 2016). However, 80% of studies evaluating CRP performance originate from high-income countries (Kapasi et al., 2016). In Southeast Asia, these evaluations are mainly hospital-based (Sutinen et al., 1998, Choo et al., 2001, Wangrangsimakul et al., 2018), with limited evidence at the community level, community-based study (Lubell et al., 2015). Good diagnostic performance of CRP in identifying bacterial infections was observed, but generalisability was limited due to demographic, clinical and diagnostic heterogeneity of these studies.

This study aimed to identify key organisms among acutely febrile children and adults attending primary health care in Southeast Asia, and to evaluate the performance of CRP for discriminating between bacteria and viruses.

## Methods

### Study sites

Chiang Rai province is the northernmost province in Thailand and borders Myanmar and Lao People’s Democratic Republic. The majority of the population are Thai, with approximately 15% ethnic minorities and hill tribes. The six participating primary care sites were located within a 30-km radius of Chiang Rai city centre, covering rural and peri-urban as well as mountainous and plateau areas.

Hlaing Tha Yar, Lower Myanmar, is a peri-urban township on the west side of Yangon. The township has the highest rates of diseases related to hygiene and environmental conditions (e.g. diarrhoea, dysentery and tuberculosis) in Yangon (Htwe et al., 2017). Four sites were included: three primary care clinics and one outpatient department from a public governmental hospital.

Both Chiang Rai and Hlaing Tha Yar are defined by a tropical climate.

### Study design

Specimens were collected from febrile patients recruited into a previously described multi-centre randomised controlled trial evaluating the impact of CRP testing on antibiotic prescription in primary care (Althaus et al., 2019). Febrile children and adults (defined as ≥12 years of age) were recruited between June 2016 and August 2017. Inclusion criteria were being aged ≥1 year with a documented fever (defined as a tympanic temperature >37.5 °C) or a chief complaint of acute fever (<14 days), regardless of previous antibiotic intake and co-morbidities other than malignancies. Exclusion criteria were symptoms requiring hospital referral, defined as: impaired consciousness; an inability to take oral medication or convulsions; a positive malaria test; the main complaint being trauma and/or injury; a suspicion of either tuberculosis, urinary tract infection, local skin infection or dental abscess; any symptom present for >14 days; any bleeding; an inability to comply with the follow-up visit at day 5.

On the day of enrolment, all patients had demographic information collected and underwent a routine clinical examination, including vital signs such as blood pressure, pulse, respiratory rate, and temperature. Patients were followed-up after their enrolment both at day 5 and day 14.

### Specimen management

**Figure 1:**
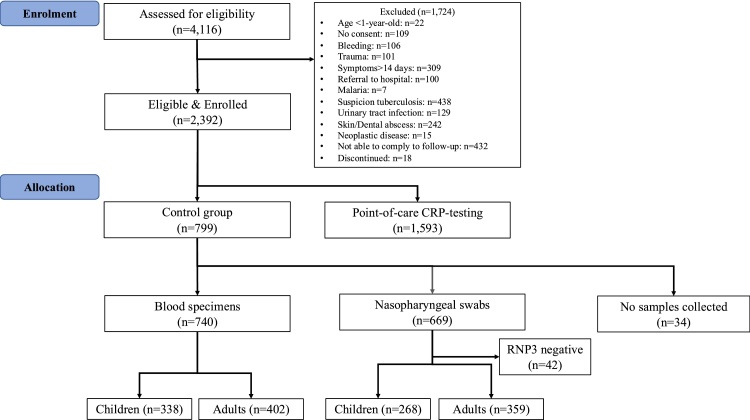
Study flowchart. RNP3, Ribonuclease P3 gene as an extraction and specimen integrity control.

Of the 2392 febrile children and adults recruited, 799 were randomly allocated to the control group with blood specimens collected for off-site CRP testing (as compared with the intervention groups that had CRP tests performed on-site). Details are illustrated in Figure 1. Antibiotics were prescribed to this group according to routine clinical practice; clinicians were not informed of the CRP results or any aetiological findings.

Microbiological investigations were carried out at Mahidol-Oxford Tropical Medicine Research Unit (MORU) in Bangkok, Thailand, and in the Amsterdam Public Health Laboratory, the Netherlands. A combination of molecular and serological assays were used to detect 35 bacteria, 30 viruses and three fungi from plasma and nasopharyngeal (NP) swab specimens (Supplementary figures and Tables 1 and 2).

Among the 740 patients for whom a blood specimen was collected, a sufficient volume of plasma was available to test 656 of them using the Rickettsial immunofluorescence assay (IFA); 626 using the bacterial singleplex polymerase chain reaction (PCR) including 16S, *Rickettsia* spp., *Orientia tsutsugamushi* and *Leptospira* spp.; and 601 using the Taqman array card (TAC) assay. The remaining plasma and the DBS collected among Myanmar children aged <5 years was used for the viral singleplex PCR on 678 patients, screening for dengue, chikungunya and zika viruses (Supplementary Table 3). CRP concentrations (mg/L) were measured on the same day of sampling using a quantitative CRP reader (NycoCard II Reader, Axis Shield, Norway), with a lower limit of detection of 8 mg/L.

### Attribution of causality

A conservative diagnostic approach was chosen, following the strength of evidence for each result: the bacteria and viruses detected in blood with the molecular and serological methods using the thresholds detailed in Table 1 were considered “target” organisms. Considering NP swabs, only influenza virus (A and B), respiratory syncytial virus (RSV), human metapneumovirus, and *Bordetella pertussis* were included as target organisms, given the higher likelihood of causality, according to available evidence in the literature (Supplementary Table 4). Other organisms detected in NP swabs were not classified as causal, because pathogenicity is not consistently demonstrated in children or adults. In case of co-detection in blood and NP swabs, target organisms detected in blood were assumed to be the primary cause of illness.

**Table 1:** Baseline characteristics of children and adults in Chiang Rai, northern Thailand and Hlaing Tha Yar, Lower Myanmar, 2016–2017.

	Children (n = 371)	Adults (n = 402)	*p*-Value
**Demographic characteristics**			
Age (in years), median (IQR)	5 (3–8)	33 (22–52)	
Female, n (%)	179 (48.3)	246 (61.2)	<0.001
Comorbidities, n (%)	11 (3.0)	111 (27.6)	<0.001
Onset of symptoms (in days), median (IQR)	2 (1–3)	3 (2–4)	<0.001
Self-reported medication intake, n (%)	232 (62.5)	290 (72.1)	0.004
Self-reported antibiotic intake, n (%)	16 (4.3)	25 (6.2)	0.237
**Clinical characteristics**			
Documented fever, n (%)	176 (47.4)	153 (38.1)	0.007
Neurological symptoms, n (%)	62 (16.7)	146 (36.3)	<0.001
Respiratory symptoms, n (%)	271 (73.1)	263 (65.4)	0.022
- URT symptoms, n (%)	249 (67.1)	240 (59.7)	0.033
Gastrointestinal symptoms, n (%)	99 (26.7)	95 (23.6)	0.328
Other symptoms, n (%)	33 (8.9)	25 (6.2)	0.170
**Biological characteristics**			
Blood specimen available, n (%)	338/371 (91.1)	402/402 (100.0)	<0.001
Total number of organisms detected in blood, n (%)	49/338 (14.5)	30/402 (7.5)	0.002
- Bacterial organisms, n (%)	18/338 (5.3)	18/402 (4.5)	0.398
- Viral organisms, n (%)	34/338 (10.1)	12/402 (3.0)	0.010
- Mixed organisms, n (%)	1/338 (0.3)	0/402 (0)	0.431
Nasopharyngeal swab available, n (%)	268/371 (72.2)	359/402 (89.3)	<0.001
Total number of swabs ≥1 organism detected, n (%)	230/268 (85.8)	238/359 (66.3)	<0.001
Total number of organisms per swab, median (IQR)	3 (1–4)	1 (0–1)	<0.001
- Bacterial organisms per swab, median (IQR)	2 (1–3)	0 (0–1)	<0.001
- Viral organisms per swab, median (IQR)	1 (0–2)	1 (0–1)	<0.001
Mixed bacterial - viral organisms in swabs, n (%)	155/268 (57.8)	57/359 (15.9)	<0.001

Comorbidities included HIV, chronic hepatitis B or C, cirrhosis, diabetes mellitus, asthma, anaemia, chronic obstructive pulmonary disease, gastritis, congenital heart or kidney disease, alcoholism, dyslipidaemia, G6PD deficiency, hypertension, rheumatic heart disease, ischaemic heart disease, thalassaemia, thyroid disease, and Alzheimer’s disease.

Neurological symptoms included headache, confusion or hearing loss.

Respiratory tract symptoms included sore throat, dyspnoea, pain on inspiration, runny nose, or cough.

URT symptoms (upper respiratory tract symptoms) were defined by the presence of either runny nose, sore throat or cough.

Gastrointestinal symptoms included nausea, vomiting, jaundice, diarrhoea, or abdominal pain.

Other symptoms were defined by the presence of fever alone or symptoms that were not neurological, respiratory or gastrointestinal. Common symptoms in this group included myalgia, arthralgia, tiredness, chills, sweating, weight loss, skin eruption, dysuria, dizziness or eye redness.

Patients were allocated to the bacterial or viral group if a bacterium or a virus was detected in blood. Considering NP swabs, only bacteria and viruses considered target were allocated to the bacterial or viral group.

### Statistical analysis

Organism distribution among children and adults (defined as ≥12 years of age) were described separately. Descriptive analysis for continuous variables with normal distribution used means and standard deviations (SD) and medians with inter-quartile ranges (IQR) for non-normally distributed continuous variables. Comparison between groups used *t*-tests for normally distributed variables, the Mann–Whitney test for non-normally distributed variables, and Chi-squared test for categorical variables.

CRP values were compared across aetiological groups and clinical syndromes using the Mann–Whitney U test for two-group comparisons and the Kruskal–Wallis test for multi-group comparisons. Non-parametric receiver operating characteristic (ROC) curves were plotted and the Wald test was used to compare areas under the curve. Covariates included patient age and prior use of antibiotics. Diagnostic accuracy was assessed by calculating the areas under the ROC curves (AUC). An AUC of >0.9 was considered excellent; 0.8–0.9, very good; 0.7–0.8, good; 0.6–0.7, average; <0.6, poor (Ying et al., 2011, Campbell, 1994). Sensitivity, specificity and percentage of correctly classified cases were also assessed for the two CRP cut-off points used in the original trial: 20 mg/L and 40 mg/L; these were compared with the accuracy of routine prescribing practice (Althaus et al., 2019). Data analyses were performed with STATA version 15 (College Station, Texas, USA).

### Ethical approval and role of the funding source

The protocol, informed consent form and case record forms were reviewed and approved by the Oxford Tropical Research Ethics Committee (OxTREC, reference 49-15), the Mahidol University Faculty of Tropical Medicine Ethics Committee (FTMEC, reference TMEC 16-015), the Myanmar Department of Medical Research (DMR, reference Ethics/DMR/2016/137), and Chiang Rai Provincial Public Health Office Research Ethics Committees (CR PHO EC, reference MO/002.17/233). This study was supported by a Wellcome Trust/ISSF grant (105605/Z/14/Z) and with the support of the Foundation for Innovative New Diagnostics (FIND) funding from the Australian government.

## Results

### Baseline characteristics

Of the 799 patients prospectively enrolled and randomised into the trial control group, 773 (96.8%) had at least one blood or NP swab specimen collected, including 371 (48.0%) children and 402 adults (52.0%). Of these 773 patients, 33 had only a NP swab and no blood collected, while 146 had only a blood specimen collected without an NP swab; among these, 80 had a blood specimen obtained on a dried blood spot (DBS). As shown in Table 1, children presented significantly earlier after symptom onset than adults, with fewer comorbidities and less self-reported medication (*p* < 0.05). Antibiotic intake declaration was similar in children and adults (*p* = 0.237).

Clinically, respiratory symptoms were the most prevalent both in children and adults. Within patients with respiratory symptoms, the most frequent were localised in the upper respiratory tract, with common cold being diagnosed in 48.2% (120/249) of children and 45.0% (108/240) of adults (*p* = 0.479). Gastrointestinal symptoms were the second most prevalent presentation, and there were no differences between children and adults.

### Organisms detected

A blood specimen was available in 91.1% (338/371) of children and all 402 adults (total n = 740). The study detected 79 (10.7%) patients with an organism in blood, more frequently in children than adults (49/338 [14.5%] *versus* 30/402 [7.5%], *p* = 0.002, respectively). This difference in detection remained significant when analysing viruses separately (*p* = 0.010), but not for bacteria (*p* = 0.398). Bacteria and virus distribution in blood is provided across age categories in Table 2: the most frequent bacteria was *Leptospira* spp., followed by *Klebsiella pneumoniae*, *Streptococcus suis* and *Rickettsia* spp. The most common virus was dengue, followed by enterovirus and rhinovirus. One child had both *Rickettsia* spp. and *Salmonella enterica* Paratyphi A, detected by the TAC assay. Corresponding patient characteristics are detailed in Supplementary Table 5.

**Table 2:** Bacterial and viral detection in blood and nasopharyngeal swabs specimens among children and adults in Chiang Rai, northern Thailand and Hlaing Tha Yar, Lower Myanmar, 2016–2017. Organisms with a high likelihood of causality in nasopharyngeal swabs are in bold.

	Blood specimens		Nasopharyngeal swabs (n = 627)	
	Children	Adults	Children (n = 268)	Adults (n = 359)
**Bacteria**				
*Aerococcus* spp. (n = 630)^a^	0	1/401 (0.3%)	–	–
*Acinetobacter baumannii*	–	–	1 (0.4%)	3 (0.8%)
*Bordetella parapertussis*	–	–	0	0
***Bordetella pertussis***	–	–	**3 (1.1%)**	**0**
*Corynebacterium diphtheriae*	–	–	1 (0.4%)	0
*Chlamydia trachomatis*	–	–	0	0
*Chlamydophila pneumoniae*	–	–	1 (0.4%)	3 (0.8%)
Group A *Streptococcus* (n = 630)	0	0	6 (2.2%)	1 (0.3%)
*Haemophilus influenzae* (n = 630)	1/229 (0.4%)	0	121 (45.2%)	42 (11.7%)
*Klebsiella pneumoniae* (n = 630)	5/229 (2.2%)	3/401 (0.8%)	13 (4.9%)	19 (5.3%)
*Leptospira* spp. (n = 634)^b^	2/232 (0.9%)	7/402 (1.7%)	–	–
*Moraxella catarrhalis*	–	–	124 (46.3%)	31 (8.6%)
*Mycoplasma pneumoniae*	–	–	1 (0.4%)	0
*Pseudomonas aeruginosa*	–	–	2 (0.8%)	1 (0.3%)
*Orientia tsutsugamushi* (n = 658)***	0	0	–	–
*Rickettsia* spp. (n = 658)^c^	3/256 (1.2%)	1/402 (0.3%)	–	–
*Salmonella* spp. (n = 630)	0	2/401 (0.5%)	–	–
*Salmonella enterica* Paratyphi A (n = 630)	2/229 (0.9%)	1/401 (0.3%)	–	–
*Staphylococcus aureus* (n = 630)	0	0	44 (16.4%)	27 (7.5%)
*Streptococcus pneumoniae* (n = 630)	0	0	130 (48.5%)	34 (9.5%)
*Streptococcus suis* (n = 630)	2/229 (0.9%)	2/401 (0.5%)	–	–
*Streptococcus* spp. (n = 630)	0	1/401 (0.3%)	–	–
**Viruses**				
Adenovirus (n = 601)^d^	0	0	13 (4.9%)	6 (1.7%)
Bocavirus (n = 601)	0	1/393 (0.3%)	12 (4.5%)	0
Chikungunya virus (n = 686)^e^	0	0	–	–
Dengue virus (n = 686)	21/290 (7.2%)	9/396 (2.3%)	–	–
Zika virus (n = 686)	0	0	–	–
Enterovirus (n = 601)	7/208 (3.4%)	1/393 (0.3%)	27 (10.1%)	16 (4.5%)
Rhinovirus (n = 601)	4/208 (1.9%)	0	82 (30.6%)	54 (15.0%)
**Influenza virus type A**	–	–	**16 (6.0%)**	**69 (19.2%)**
**Influenza virus type B**	–	–	**13 (4.9%)**	**9 (2.5%)**
Human coronavirus	–	–	20 (7.5%)	17 (4.7%)
Measles (n = 601)	0	0	1 (0.4%)	1 (0.3%)
Parainfluenza virus (1-3)	–	–	19 (7.1%)	5 (1.4%)
**Human metapneumovirus**	–	–	**12 (4.5%)**	**4 (1.1%)**
Rubella virus (n = 601)	0	1/393 (0.3%)	–	–
**Respiratory syncytial virus**	–	–	12 (4.5%)	12 (3.3%)
Cytomegalovirus	–	–	44 (16.4%)	4 (1.1%)
Varicella-Zoster virus (n = 601)	2/208 (1.0%)	0	3 (1.1%)	0

a 630 blood specimens tested for bacterial screening, using the Taqman array card (TAC) assay (n = 601) and bacterial singleplex polymerase chain reaction (PCR) assay (n = 626).

b 634 blood specimens tested for *Leptospira* spp. screening, using the TAC assay (n = 601), the bacterial singleplex PCR assay (n = 626) and the microagglutination test (n = 134).

c 658 blood specimens tested for *Orientia tsutsugamushi* and *Rickettsia* spp. screening, using the TAC assay (n = 601), the bacterial singleplex PCR assay (n = 626), and the indirect immunofluorescence assay (n = 656).

d 601 blood specimens tested using the TAC assay only (n = 601).

e 686 blood specimens tested for dengue, chikungunya and zika virus screening, using the TAC assay (n = 601), the viral singleplex PCR assay on fresh blood (n = 626) and dried blood spot (n = 245).

Nasopharyngeal swabs were available in 268/371 (72.2%) children and 359/402 (89.3%) adults. Organisms were more frequently detected and with a higher median number in children than adults (230/268 [85.8%] *versus* 238/359 [66.3%] and 3 [IQR 1–4] *versus* 1 [0–1], *p* < 0.001), respectively. The number of organisms per swab remained higher among children when analysing bacteria and viruses separately (*p* < 0.001). Similarly, co-detection of bacteria and viruses was more frequent in children than adults (155/268 [57.8%] *versus* 57/359 [15.9%], *p* < 0.001, respectively).

Among children, the most prevalent organism was *Streptococcus pneumoniae* (*S. pneumoniae*), detected in 48.5% of cases, followed by *Moraxella catarrhalis* (46.3%), *Haemophilus influenzae* (45.2%) and rhinovirus (30.6%). Among adults, the most prevalent organism detected was influenza virus type A (19.8%), followed by rhinovirus (15.0%), *H. influenzae* (11.7%) and *S. pneumoniae* (9.5%). The distribution of bacteria and viruses per age category is illustrated in Supplementary Figure 3, as well as co-detections.

Among all organisms detected in NP swabs, target bacteria and viruses were found among 177 of 627 patients (28.2%), including 67 of 268 children (25.0%) and 110 of 359 (30.6%) adults. Influenza virus type A was the most prevalent target organism detected in 85 of 627 NP swabs (13.6%), followed by RSV detected in 24 cases (3.8%), Influenza virus type B in 22 cases (3.5%), hMPV in 16 cases (2.6%), and *Bordetella pertussis* in three paediatric cases (0.5%).

### Patient outcomes by aetiological group

No evidence for a difference in antibiotic prescription was observed between the bacterial and viral groups at day 0, and clinical outcomes were also not significantly different between the two groups (Table 3).

**Table 3:** Outcome characteristics by aetiological group in Chiang Rai, northern Thailand and Hlaing Tha Yar, Lower Myanmar, 2016–2017.

Outcome characteristics	Bacteria (n = 36)	Viruses (n = 191)	*p*-Value
Antibiotic prescription at day 0, n (%)	12 (33.3)	76 (39.8)	0.466
- Broad-spectrum antibiotic at day 0, n (%)	1/12 (8.3)	8/76 (10.5)	0.795
Antibiotic prescription from day 0-14, n (%)	14 (38.9)	79 (41.4)	0.782
- Broad-spectrum antibiotic from day 0-14, n (%)	2/14 (14.3)	8/79 (10.1)	0.621
Symptom resolution at day 5, n (%)	23 (63.9)	115 (60.2)	0.519
Symptom severity at day 5, median (IQR)	1 (1–1)	1 (1–1)	0.226
Documented fever at day 5, n (%)	2 (5.6)	3 (1.6)	0.123
Elevated CRP at day 5, n (%)	1 (2.8)	3 (1.6)	0.592
Symptom resolution at day 14, n (%)	35 (97.2)	180 (94.2)	0.245
Symptom severity at day 14, median (IQR)	1 (1–1)	1 (1–2)	0.103
Documented fever at day 14, n (%)	0 (0)	1 (0.5)	0.665
Occurrence of SAE, n (%)	0 (0)	0 (0)	1.000
Unscheduled visits, n (%)	0 (0)	6 (3.1)	0.281

The prescription of antibiotics at the facility was considered between enrolment on day 0 until day 14 of follow-up.

Severity was ranked from 1 to 4 with severity = 1 as the less severe presentation.

CRP: C-reactive protein.

Elevated CRP defined as ≥50 mg/L in children and ≥100 mg/L in adults.

SAE: serious adverse event, defined as admission to hospital or death within 14 days of enrolment.

Broad-spectrum antibiotics included ceftriaxone, cefixime, ciprofloxacin, levofloxacin, azithromycin, and amoxicillin with clavulanic acid.

Among patients with a bacterial organism, two-thirds did not receive any antibiotic (24/36, 66.7%), and those antibiotics received were often ineffective for the detected bacteria (Supplementary Table 6). Of the 12 patients with either *Leptospira* spp. or *Rickettsia* genus, nine (75%) did not receive any antibiotic. No evidence for a difference in clinical outcomes was observed after 14 days of follow-up, regardless of whether an antibiotic was prescribed.

### Accuracy of current prescribing practices and CRP-guided treatment

**Figure 2:**
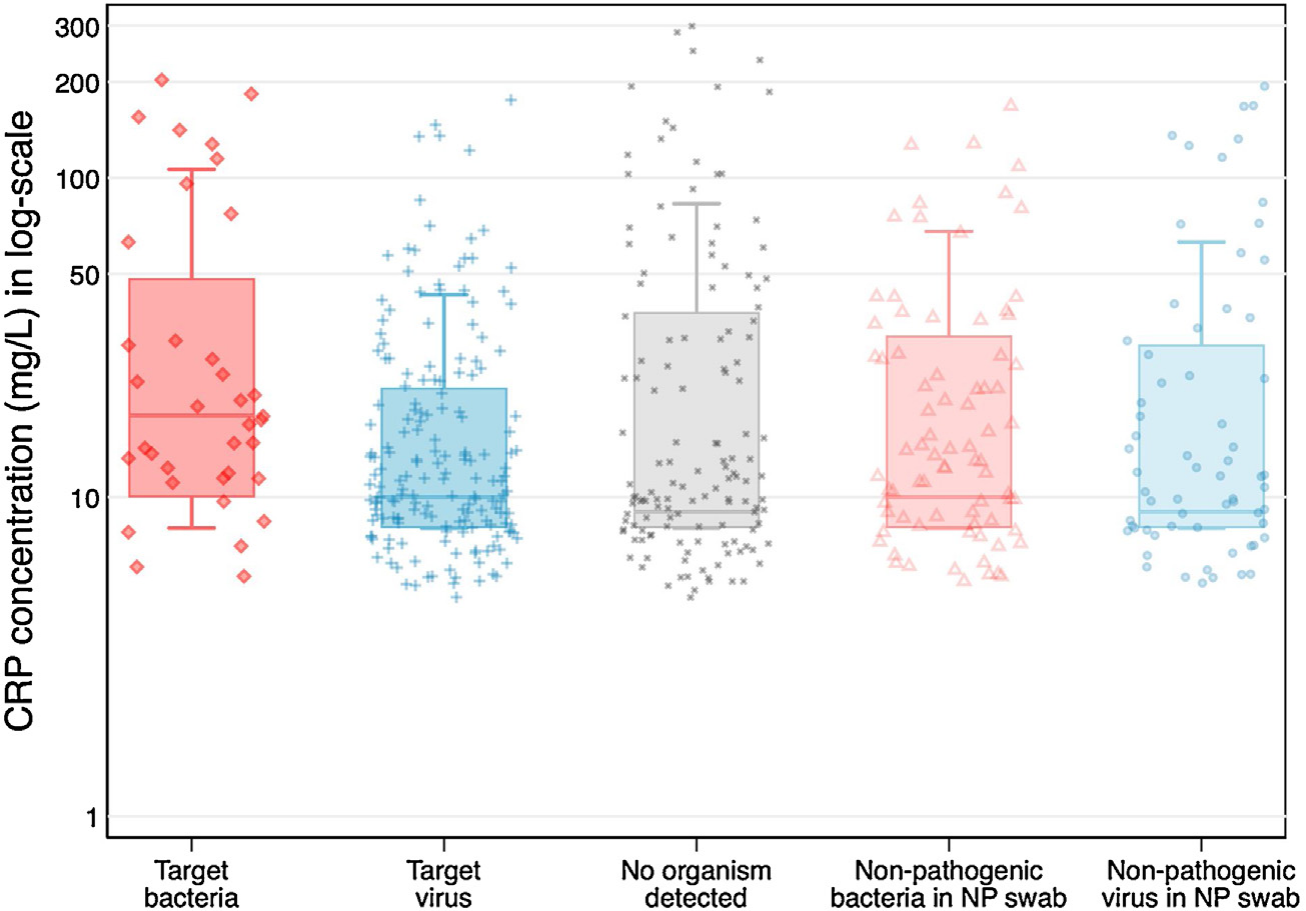
C-reactive protein (CRP) concentration (mg/L) using a log scale per aetiological group in Chiang Rai, northern Thailand and Hlaing Tha Yar, Lower Myanmar, 2016–2017. CRP concentrations (all in log-scale) are coloured in blue for viruses, red for bacteria and in grey when no organisms were detected neither in blood specimens nor in nasopharyngeal (NP) swabs. Box boundaries show 25th and 75th percentiles of CRP concentrations and lines within the boxes show the medians. The whiskers indicate the 10th and 90th percentile of CRP concentrations.

Of the 227 patients with a target organism detected, 36 (15.9%) were allocated to the bacterial group and 191 (84.1%) to the viral group. The median CRP concentration was higher in the bacterial group compared with the viral one (18 [10–49] mg/L *versus* 10 [≤8–22] mg/L, *p* = 0.003, respectively). Among the 422 patients for whom no organism was detected or the organism was not considered causal, the median CRP was 10 (≤8–29) mg/L lower than among patients with a target bacteria (*p* = 0.004) but similar to patients with a target viral organism (*p* = 0.826). The distribution of CRP concentrations is presented by aetiological group in Figure 2.

**Figure 3:**
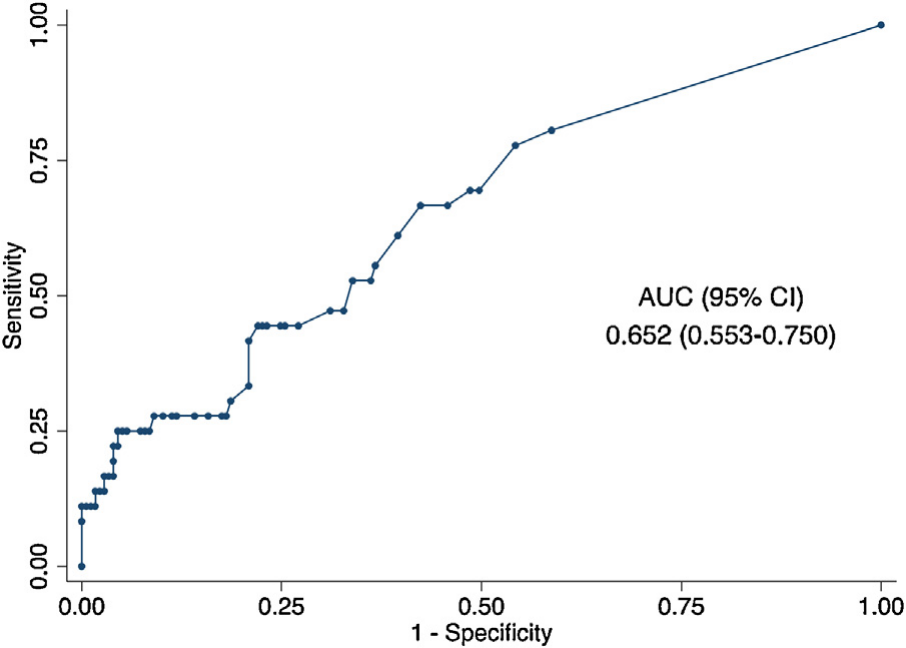
Diagnostic accuracy of C-reactive protein (CRP)-testing for distinguishing bacterial from viral targeted organisms in Chiang Rai, northern Thailand and Hlaing Tha Yar, Lower Myanmar, 2016–2017.

The AUC for CRP distinguishing bacterial from viral target organisms was 0.652, 95% confidence interval (0.553–0.750), classified as average, as illustrated in Figure 3.

In the absence of CRP-testing, the sensitivity of current prescribing practices for detecting bacterial target organisms was 33.3% (95% CI 18.6–51.0%) and specificity was 60.2% (95% CI 52.9–67.2%). Using a threshold of 20 mg/L would imply an increase in sensitivity to 47.2% (95% CI 30.4–64.5%) and an increase in specificity to 68.9% (62.3–75.5%). Use of the higher threshold of 40 mg/L would imply a lower sensitivity of 27.8% (14.2–45.2%) and a higher specificity of 88.7% (83.3–92.6%). In total, based on current prescribing practices during the study, 57.8% of patients were correctly classified for the prescription of an antibiotic, while CRP-guided treatment would have increased this to 65.8% using a 20 mg/L threshold and to 79.3% using a 40 mg/L threshold.

## Discussion

This study investigated the spectrum of organisms among febrile children and adults in the community and evaluated the performance of CRP in distinguishing bacteria from viruses, including its potential impact on antibiotic prescription compared with current practice. Patients were prospectively recruited across ten sites in Thailand and Myanmar, including urban, semi-urban and rural areas, spanning over a full calendar year.

*Leptospira* spp., influenza virus, RSV and dengue virus were the leading organisms identified, which is consistent with previous reports in the region (Mueller et al., 2014, Lubell et al., 2015). The broad inclusion criteria, allowing for enrolment of all patients aged >1 year regardless of previous antibiotic intake, comorbidities (apart from malignancy), or clinical presentation, made the findings more generalisable than previous studies.

Investigating non-malarial acute febrile illness remains challenging in resource-poor areas (Mueller et al., 2014), and despite screening for multiple organisms on blood and respiratory specimens, it was only able to identify a probable cause of fever in 227 (29.4%) of patients. This low detection may be explained by the inclusion of only non-severe outpatients (Hackett et al., 2002, Jiang et al., 2017, Reimer et al., 1997), while other studies in Southeast Asia recruiting more severe and hospitalised patients identified an organism in around 50% of cases (Chheng et al., 2013, Mayxay et al., 2015, Southeast Asia Infectious Disease Clinical Research Network, 2017, Wangrangsimakul et al., 2018). Of the detected organisms, 15.9% (36/227) were bacteria, which may be explained by the lower risk of bacterial infections in non-severely ill patients, and where present, characterised by lower bacterial loads (Reimer et al., 1997).

Most bacteria were identified using a singleplex PCR and not the TAC assay, while viruses were equally detected by these two molecular methods. This lower sensitivity in the TAC assay for the detection of bacteria has been described in previous studies using multi-pathogen molecular detection platforms (Warhurst et al., 2015, Weller et al., 2012). The trade-off between advantages for screening multiple organisms at the same time with a simplified molecular platform should be weighed against potentially lower sensitivity, especially for bacteria such as *O. tsutsugamushi* or *Leptospira* spp., which are considered important drivers of acute febrile illness in Southeast Asia (Xu et al., 2017, Laras et al., 2002).

Conversely, the use of multiplex assays runs the risk of detecting organisms for which the causality and interpretation can be challenging: this study detected *Klebsiella pneumoniae* in the blood of eight patients, of whom six did not receive any antibiotic. It is unknown whether these patients had a transient self-limiting infection, carriage, or were false positive. A 2018 community-based study also used the TAC assay to identify infections among neonates in South Asia, but detected the presence of certain organisms among both controls and cases (Saha et al., 2018). A 2019 multi-country study into causes of severe pneumonia also excluded molecular assay results positive for *Klebsiella pneumoniae* because of poor assay specificity (O’Brien et al., 2019).

Furthermore, most of the patients presented with low CRP regardless of whether a bacterial or viral organism was detected, and recovered regardless of whether an antibiotic was prescribed. It is likely that invasive bacterial infections requiring an antibiotic are exceptions, while most primary care patients present with a self-limiting infection (Van den Bruel et al., 2007). Other primary care-based studies have recently supported restriction of antibiotic prescription to a small minority of patients: in Tanzania, a clinical trial using a 80 mg/L threshold lowered antibiotic prescription to 2.3% without affecting outcomes, while a study from the USA concluded that 72% of outpatients attending a general practice for a respiratory presentation should not even require a medical consultation, let alone an antibiotic prescription (Keitel et al., 2019, Renati and Linder, 2016). Strategies whereby testing for CRP as a predictor of clinical outcome rather than determining aetiology have been evaluated in primary care: a cluster-randomised controlled trial in Belgium showed CRP to rule-out serious infection using a 5 mg/L threshold, while a systematic review found CRP testing to be useful in identifying serious infections among febrile children (Verbakel et al., 2016, Van den Bruel et al., 2011).

In the current study, CRP performance in distinguishing bacteria from viruses was average (AUC 0.65) and lower than another study from the same region, which found AUC 0.83 among 1372 patients with a microbiologically-confirmed diagnosis from Thailand, Cambodia and Lao PDR (Lubell et al., 2015). It is likely that most bacteria detected in the current study caused a self-limiting infection, while previous CRP evaluations included more severe and hospitalised patients with bacteraemia detected by blood culture. Despite average performance, CRP-guided treatment would still result in more accurate classification of fever as bacterial or viral aetiology compared with current practices (assuming that prescription of antibiotics was indicative of health workers believing the illness to be of bacterial origin). This supports the findings from the original trial, where CRP testing safely reduced antibiotic prescription, which are in line with similar primary care-based studies both in Asia and Africa (Keitel et al., 2019, Keitel et al., 2017, Do et al., 2016).

This study had several limitations mostly relating to the limited scope and accuracy of the reference diagnostic tests, and the impact of even slightly less than perfect “gold-standard” reference tests on the evaluation of new diagnostic and biomarker tests can be profound (McHugh et al., 2019, Waikar et al., 2013). As mentioned above, the sensitivity and specificity of the multiplex TAC assay was not optimal for bacteria detection, and the absence of convalescent specimen impeded the ability to diagnose patients based on serology, particularly with respect to bacterial zoonoses. Even genuine detection of bacterial and viral DNA in normally sterile sites cannot be used to conclusively determine causality, as this has been reported among healthy individuals, and in patients even weeks after recovery from infections, challenging the interpretation of molecular assays (Williams et al., 2014, Decker et al., 1996). Blood culture was not available and this further limited the aetiological investigation. The current study did not recruit a concomitant control group, which precludes robust attribution of causality in the organisms that were detected, particularly in NP swabs. In a paediatric study in Asia and Africa, the inclusion of controls matched with pneumonia cases weakened the evidence of causality for almost all detected organisms (O’Brien et al., 2019), and only RSV, hMPV, influenza virus A and B, parainfluenza virus type 1, and *B. pertussis* were considered pathogenic, which is consistent with other LMIC-based studies (Bénet et al., 2017, Pretorius et al., 2016). These organisms, however, are sometimes present in healthy individuals, with a prevalence of influenza virus among healthy children between 0–6%, RSV at 0–9% and hMPV between 0–7% (Bosch et al., 2013). On the other hand, the current study did not regard other organisms detected in NP swabs such as rhinovirus, parainfluenza virus or *S. pneumoniae* as pathogenic, because these are commonly detected among healthy individuals (O’Brien et al., 2019, Jansen et al., 2011, Birger et al., 2018). All these limitations in reference diagnostic tests might explain the average performance of CRP in the current analysis.

## Conclusion

This study presented the key organisms detected among febrile children and adults attending primary health care in Southeast Asia. The performance of CRP in distinguishing between bacterial and viral organisms was limited, although the findings suggest that CRP-guided treatment would increase the appropriate use of antibiotics with respect to aetiology. This is supported by the overall reduction in prescribing compared with current practice demonstrated in the original trial. The findings also support conclusions from previous studies that even in the presence of bacterial organisms, very few ambulatory patients are likely to benefit from the extensive and poorly targeted antibiotic prescribing practices that currently prevail in most Southeast Asian primary care settings.

## Contributors

TA, JT, PWS, TW, NPJD, FS, PT, SD, SB, JMW, MHD and YL contributed to the literature review. The study was designed by TA, RCG, TW, NPJD, FS, PWS, and YL. Data collection was coordinated by MMMS, RCG and JT. Data analysis by TA, JT, PP, PT, JMW, MHD and YL, and data interpretation by TA, JT, PWS, TW, PP, FS, PT and YL. Figures were created by TA, PP, PT and YL. The manuscript was written by TA, PT, and YL. All authors reviewed the manuscript, added appropriate revisions, agreed to submission for publication, and approved the final version. TA and YL are guarantors.

## Declaration of interest

The funders had no role in study design, data collection, data interpretation or writing the manuscript. The corresponding author had full access to all the data and took the final decision to submit for publication.
