# Supplementary material for: Causes of fever in primary care in Southeast Asia and the performance of C-reactive protein in discriminating bacterial from viral pathogens
Source: Int J Infect Dis. 2020 Jul;96:334–42. doi: 10.1016/j.ijid.2020.05.016 (PMC7211754; doi:10.1016/j.ijid.2020.05.016)
Supplement: Supplementary file 1 [file mmc1.docx]

**Causes of fever in primary care in Southeast Asia and performance of C-reactive protein for discriminating bacterial from viral pathogens**

**Supplementary material**

**Supplementary figure 1.** Diagnostic tests by type of specimen

IFA – Indirect immunofluoresence assay

MAT – Microagglutination test

PCR - Polymerase chain reaction

RNP3 – Ribonuclease protein 3

**Supplementary figure 2 A.** Configuration of the TaqMan Array Card for detection of the organisms in blood specimens

**Supplementary figure 2 B.** Configuration of the TaqMan Array Card for detection of the organisms in nasopharyngeal swabs.

**Supplementary figure 3.** Network analysis for bacterial and viral detection and combination in nasopharyngeal swabs among children and adults in Chiang Rai, northern Thailand and Hlaing Tha Yar, Lower Myanmar, 2016-2017.


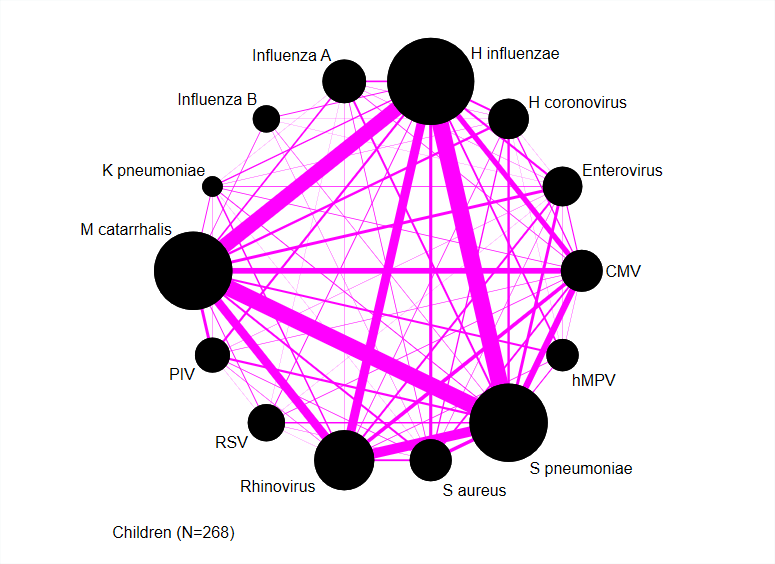

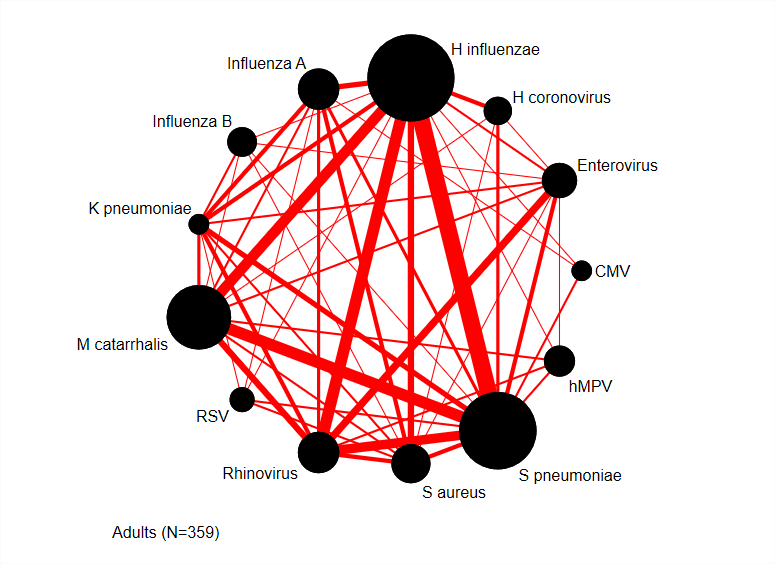


The size of the black dot represents the frequency of organism detection in nasopharyngeal swabs. Only organisms detected in more than 10 specimens are represented.

Each of the axes between the dots represents the frequency of combination between two organisms.

Bacterial organisms: *K pneumoniae* is *Klebsiella pneumoniae; S pneumoniae* is *Streptococcus pneumoniae; M catarrhalis* is *Moraxella catarrhalis; S aureus* is *Staphylococcus aureus;* and *H influenzae* is *Haemophilus influenzae.*

Viral organisms: CMV is cytomegalovirus; H coronavirus is human coronavirus (229E; NL63; OC43 and HKU1); hMPV is human metapneumovirus; RSV is respiratory syncytial virus; and PIV is parainfluenza virus (type 1-3).

**Supplementary table 1.** Details of microbiological management

| *Sample collection*  Blood specimens were collected using ethylenediamine tetra-acetic acid (EDTA) tubes (Vacuette EDTA K3 tube, Greiner Bio-One International GmbH, Austria) in each primary care centre and transported in a cool box to a local laboratory. Plasma was obtained after centrifugation on the day of collection and stored at -80˚C until analysis. A dried blood spot (DBS, Whatman 31ET CHR, Merck, Germany) was obtained from capillary specimens when venous blood was not collected, primarily in children under 5 in Myanmar.  In addition to blood specimens, patients from the control group had a nasopharyngeal (NP) swab, using the Sigma VCM^®^ (Wiltshire, England), which includes a cellular foam bud and a transport medium suitable for nucleic acid preservation and molecular amplification [1]. The study staff were trained for the swab procedure according to the WHO standards (<https://www.who.int/influenza/rsv/rsv_collection_transport_storage_samples/en/>). Swabs were then stored at in a cool box after collection, and transported to a local laboratory on the same day for aliquoting and storage at -80˚C. |
| --- |
| *Total nucleic acid preparation for the Taqman Array Card assay* |
| The total nucleic acid (TNA) was prepared from each 350 µl of plasma or NP swab using the MagNA Pure Compact Extractor (Roche Applied Sciences, Indianapolis, IN, USA), following manufacturer’s instructions. For the pre-lysis step, Bacterial Lysis Buffer (BLB) (Roche, Germany) and proteinase K were added at the same specimen volume and 10% of the specimen volume, respectively. |
| *Taqman Array Card assay procedure* |
| The TaqMan Array Card (TAC) (Life Technologies, Foster City, USA) is a multiple-pathogen detection method based on real-time PCR evaluated in detecting various respiratory, enteric pathogens in children and adults from both high income countries and LMICs [2-5].  TAC offers several advantages: i) the panel of pathogens targeted can be customized, ii) a minimal volume of specimen is sufficient to test for up to 48 targets, iii) each card test for six specimens at the same time, iv) only general laboratory skills needed, v) positive and negative controls in each card and within each patient ensure the quality of the PCR reaction, and vi) low risk of contamination due to the hermetic well plate format.  In this study, we customised the blood TAC assay to target nineteen bacteria, fourteen viruses and two parasites, as illustrated in supplementary figure 2 A.  In addition, there were two extrinsic controls: one with the human RNase P (RNP3) gene as an extraction and specimen integrity control, and another one as an internal positive control for the amplification (IPCO) [6-8]. For NP swabs, we customized the respiratory TAC assay to target sixteen viruses, sixteen bacteria and one fungus, as illustrated in supplementary figure 2 B. Micro-organisms were selected in the panel according to those commonly found in Southeast Asia [9-14].  All assays were performed in duplicate to maximise sensitivity, and a coefficient of variation between the duplicates was calculated to ensure the TAC measurement consistency (supplementary table 2). In addition to the 6 channels for patient’s specimen, one channel with sterile water was used as a negative control ensuring the absence of contamination in the PCR reaction, and one channel with TAC positive control, with single-use aliquots of combined RNA transcript for positive amplification of all targets on all TAC formats, provided by CDC.  Each 50 µl of patient TNA, or negative control, or positive control was mixed with 50 µl of Quanta qScript XLT One-step RT-qPCR ToughMix, low ROX (Quanta Biosciences, USA). The ToughMix enzyme mix contains all reagents, including the polymerase and reverse transcriptase enzymes, and has been demonstrated to enhance stability during PCR reactions with storage requiring refrigeration temperatures only [2].  Cards were centrifuged to homogeneously distribute the reaction mix to all wells (1 minute at 1,200 rpm twice) and sealed, following the manufacturer’s instructions. Cards were run on the ViiA 7^TM^ real-time PCR system (Life Technologies) using PCR cycling conditions comprising 10 min at 50˚C and 20 s at 95˚C followed by 45 two-step cycles of 3 s at 95˚C and 30 s at 60˚C as described previously [15]. |
| *Singleplex PCR assay* |
| Dengue, chikungunya and zika viruses were further screened by a real-time RT-PCR assay, using the TaqMan^®^ Fast Virus One-Step RT-PCR Master Mix (Applied Biosystems, Foster City, California) as described previously [16]. Performance of real-time RT-PCR on DBS for detecting these three arboviruses has been validated when compared with blood specimens [17].  Three probe-based real-time PCR assays further investigated *O. tsutsugamushi* (47 kDa *htrA* gene), *Rickettsia* spp. (17 kDa gene) as well as pathogenic *Leptospira* spp. targeting the *rrs* gene, as described previously [18-21]. We also screened for 16S RNA using an in-house real-time PCR targeting the 16S rRNA region V1 to V3 [22, 23]. For positive PCR, the PCR products were undertaken for Sanger DNA sequencing (Macrogen, Korea). We blast the resulted 16S rRNA sequences to NCBI data, OTU equal to or more than 97%. |
| *Extraction for the singleplex PCR assay* |
| We used an in-house DNA extraction method (Gentra Puregene Blood kit, Qiagen, Norway) as we previously published, we used each 1 mL of whole blood for nucleic acid preparation. 3 mL of red blood cell (RBC) lysis solution was added to the whole blood and centrifuged at 3,000 rpm for two minutes [11]. After discarding the supernatant, the remaining liquid was mixed with 1mL of cell lysis solution and 5 µL of RNase mixture. We added 333 μL protein precipitation solution (Gentra Puregene Blood kit, Qiagen, Norway), centrifuged at 3,000 rpm for 6 minutes and transferred the supernatant into a tube with undiluted isopropanol using a pasture pipette. We then centrifuged and discarded the supernatant, added 1 mL of 70% ethanol and inverted twice to wash the DNA pellet. Then, we centrifuged at 3,000 rpm for 1 minute at 25˚C, discarded the supernatant, centrifuged again at 3,000 rpm for 10 seconds and discarded all the solution using fine tip pasture pipette. We dried the pellet by leaving the tube open for 5 minutes, added a 100 μL preheated DNA Hydration Solution (Gentra Puregene Blood kit, Qiagen, Norway) at 65˚C, mixed and incubated at 65˚C for 1 hour. |
| *Serology* |
| Leptospirosis was also screened by IgM antibodies detection on a single acute specimen using a commercially available *Leptospira* IgM ELISA (Panbio Pty., Ltd., Queensland, Australia). *Leptospira* IgM ELISA positive specimens were then confirmed by microscopic agglutination test (MAT), in microtiter plates and using reference strains of 24 *Leptospira* serovars [24]. The serum titre was defined as the final dilution that showed 50% agglutination. Reciprocal agglutination titres of greater than or equal to 50% were considered positive reactions. We regarded leptospiral MAT as positive for a titre ≥1:800 following the 2013 CDC recommendations (<https://wwwn.cdc.gov/nndss/conditions/leptospirosis/case-definition/2013/>).  Scrub typhus, typhus group (TG) and spotted fever group rickettsiosis (SFG) were also screened on a single acute specimen by IgM ELISA (InBios International Inc., Seattle WA, USA). Scrub typhus was serologically confirmed by indirect immunofluorescence assay (IFA) using slides coated with O. tsutsugamushi (strains Karp, Kato, and Gilliam) as previously described [25]. A stringent diagnostic positivity criterion was an admission IgM titre ≥1:3200 [26]. Typhus group and SFG were only considered probable cases using IFA by a single titre to ≥1:400, as no paired specimen was available [27]. |

**Supplementary table 2.** Diagnostic tests performed for bacterial, viral and fungal organisms

| **Blood specimens** | **Specimen** | **Assay^*^** | **Comments on diagnostic tests** |
| --- | --- | --- | --- |
| *Leptospira* spp. | DNA from plasma | TAC | All serovars of all species from all genus |
|  | DNA from plasma | Singleplex PCR | Real-time PCR targeting *rrs* gene [21] |
|  | Plasma | ELISA | *Leptospira* IgM ELISA (Panbio Pty., Ltd., Queensland, Australia) [28] |
|  | Plasma | MAT^**^ | Using the following 24 serovars representing strains from *Leptospira interrogans*: Australis, Autumnalis, Ballum, Bataviae, Canicola, Cellidoni, Cynopteri, Djasiman, Grippotyphosa, Hebdomadis. Icterohaemorrhagiae, Javanica, Louisiana, Manhao, Mini, Panama, Pomona, Pyrogenes, Ranarum, Sarmin, Sejroe, Shermani, Tarasovi, Semaranga [29] |
| *Orientia tsutsugamushi* | DNA from plasma | TAC | Real-time PCR targeting the 47 kDa outer membrane protein of *Orientia tsutsugamushi* [30] |
|  | DNA from plasma | Singleplex PCR | Real-time PCR targeting the 47 kDa outer membrane protein of *Orientia tsutsugamushi* [30] |
|  | Plasma | IFA | IFA of IgM against *Orientia tsutsugamushi* [31] |
| *Rickettsia* spp. | DNA from plasma | Singleplex PCR | Real-time PCR targeting 17 kDa antigen for the genus *Rickettsia* [30, 32] |
| *Rickettsia typhi* | Plasma | IFA | IFA of IgM against *Rickettsia typhi* [31] |
| Spotted fever group | Plasma | IFA | IFA of IgM against *Rickettsia conorii,*  *Rickettsia rickettsii* or *Rickettsia australis* or *Rickettsia helvetica* [33] |
| Rubeola virus | DNA from plasma | TAC | All subtypes of measles virus |
| Rubella virus | DNA from plasma | TAC |  |
| Parechovirus | DNA from plasma | TAC | All genotypes |
| Japanese encephalitis virus | DNA from plasma | TAC |  |
| Dengue virus | DNA from plasma | TAC | Serovars 1-4 |
|  |  | Singleplex PCR | Real-time PCR [16] |
| Chikungunya virus | DNA from plasma | TAC |  |
|  |  | Singleplex PCR | Real-time PCR [16] |
| Zika virus | DNA from plasma | TAC |  |
|  |  | Singleplex PCR | Real-time PCR [16] |
| Varicella-zoster virus | DNA from plasma | TAC |  |
| Enterovirus | DNA from plasma | TAC | All serotypes |
| Rhinovirus | DNA from plasma | TAC |  |
| Bocavirus | DNA from plasma | TAC |  |
| Nipah virus | DNA from plasma | TAC |  |
| *Salmonella enterica* Paratyphi A | DNA from plasma | TAC |  |
| *Salmonella* spp. | DNA from plasma | TAC |  |
| *Escherichia coli/ Shigella* spp. | DNA from plasma | TAC | Does not detect *Shigella dysenteriae* type 1 |
| *Klebsiella pneumoniae* | DNA from plasma | TAC |  |
| *Haemophilus influenzae* type B | DNA from plasma | TAC | May cross-react with *Haemophilus influenzae* type A |
| *Streptococcus suis* | DNA from plasma | TAC |  |
| Group A *Streptococcus* | DNA from plasma | TAC |  |
| Group B *Streptococcus* | DNA from plasma | TAC |  |
| *Bartonella* spp. | DNA from plasma | TAC |  |
| *Burkholderia pseudomallei* | DNA from plasma | TAC |  |
| *Brucella* spp. | DNA from plasma | TAC |  |
| *Staphylococcus aureus* | DNA from plasma | TAC |  |
| *Yersinia* spp. | DNA from plasma | TAC |  |
| *Plasmodium falciparum & vivax* | DNA from plasma | TAC |  |
| **Respiratory specimens** |  |  |  |
| Viruses | NP swabs | TAC | Influenza (A & B), Adenovirus (all serotypes except 40 & 41), Enterovirus, Respiratory syncytial virus (A & B), human metapneumovirus, Rhinovirus, Parainfluenza virus (1-3), Coronavirus (NL63, HKU1, 229E, OC43), MERS Coronovirus, Bocavirus, Cytomegalovirus, Hepatitis E virus (all genotypes), Rubella virus, Varicella-zoster virus and Parechovirus |
| Bacteria | NP swabs | TAC | *Mycoplasma pneumoniae*, *Chlamydophila pneumoniae*, *Moraxella catarrhalis;* *Bordetella pertussis, Bordetella parapertussis, Pseudomonas aeruginosa, Acinetobacter baumannii, Burkholderia pseudomallei, Chlamydia trachomatis*, *Klebsiella pneumoniae,* *Leptospira* spp., *Escherichia* *coli*, *Shigella* spp., *Streptococcus pneumoniae*, group A *Streptococcus*, *Staphylococcus aureus, Corynebacterium diphtheriae* and *Haemophilus influenzae* |
| Fungi | NP swabs | TAC | *Pneumocystis jirovecii* |

*Molecular assays using PCR: polymerase chain reaction, serology assays including MAT: micro-agglutination test; IFA: immunofluorescence antibody assay; ELISA: enzyme-linked immunosorbent assay; NP: nasopharyngeal; TAC: Taqman array card

**Microagglutination test was only carried out in specimens positives for the *Leptospira* immunofluorescence antibody assay (immunoglobulin M ≥11 Panbio units)

**Supplementary table 3.** Diagnostic methods: number of testing and positives in total, in children and in adults.

|  | **Blood specimens** | | | | | **Nasopharyngeal swabs** | |
| --- | --- | --- | --- | --- | --- | --- | --- |
|  | **Bacterial singleplex**  **PCR** | **Viral singleplex**  **PCR** | **Taqman Array Card** | ***Rickettsia* genus IFA** | ***Leptospira* spp.**  **MAT** | **Taqman Array**  **Card** |  |
| **Total** | Tested: 626  Positive: 11 (1.8%) | Tested: 678  Positive: 26 (3.8%) | Tested: 601  Positive: 63 (10.1%) | Tested: 656  Positive: 1 (0.2%) | Tested: 154  Positive: 2 (1.3%) | Tested: 627  Positive: 468 (74.6%) |  |
| **Children** | Tested: 225  Positive: 5 (2.2%) | Tested: 283  Positive: 18 (6.4%) | Tested: 208  Positive: 26 (12.5%) | Tested: 254  Positive: none | Tested: 55  Positive: none | Tested: 268  Positive: 230 (85.8%) |  |
| **Adults** | Tested: 401  Positive: 6 (1.6%) | Tested: 395  Positive: 8 (2.0%) | Tested: 393  Positive: 13 (3.3%) | Tested: 402  Positive: 1 (0.3%) | Tested: 99  Positive: 2 (2.0%) | Tested: 359  Positive: 238 (66.3%) |  |

Bacterial singleplex polymerase chain reaction (PCR) included 16S, *O. tsutsugamushi, Rickettsia* genus and *Leptospira* spp.

IFA: immunofluorescence assay

MAT: microagglutination test

NP swabs: nasopharyngeal swabs

PCR: polymerase chain reaction

Viral singleplex PCR included dengue, chikungunya and zika virus

**Supplementary table 4.** Evidence review for classifying influenza virus A & B, respiratory syncytial virus, human metapneumovirus and *Bordetella pertussis* as causal in nasopharyngeal swabs

| **Organism** | **Methods** | **Findings** | **References** |
| --- | --- | --- | --- |
| Influenza virus  A & B | Multi-site international case-control study among 1-59-month-old children with respiratory symptoms | OR 3.6, 95% CI (2.4-5.3) | [34] |
|  | Multi-site international case-control study among 0-59-day-old babies with community-acquired serious infections | Influenza A: OR 1.5, 95% CI (0.8-2.7)  Influenza B: OR 3.8, 95% CI (1.2-12.4) | [35] |
|  | Multi-site case-control study among children and adults with ILI | Children: RRR 24.0, 95% CI (9.5-60.7)  Adults: RRR 12.3, 95% CI (7.5-20.3) | [36] |
|  | Nested case-control study among children 0-42-month-old with pneumonia | OR 4.13, 95% CI (2.06-8.26) | [37] |
| Respiratory syncytial virus (RSV) | Multi-site international case-control study among 1-59-month-old children with respiratory symptoms | OR 14.0, 95% CI (11.4-17.1) | [34] |
|  | Multi-site international case-control study among 0-59-day-old babies with community-acquired serious infections | OR 6.3, 95% CI (4.2-9.4) | [35] |
|  | Multi-site case-control study among children and adults with SARI | Children: RRR 9.9, 95% CI (6.2-15.8)  Adults: RRR 2.2, 95% CI (1.2-3.9) | [36] |
|  | Nested case-control study among children 0-42-month-old with pneumonia | OR 8.05, 95% CI (4.21-15.38) | [37] |
|  | Multi-site case-control study among children and adults with a community-acquired pneumonia | AF 93%, 95% CI (87-97%)  OR 15.2, 95% CI (7.92-29.2) | [38] |
| Human metapneumovirus (hMPV) | Multi-site international case-control study among 1-59-month-old children with respiratory symptoms | OR 6.3, 95% CI (4.8-8.2) | [34] |
|  | Multi-site international case-control study among 0-59-day-old babies with community-acquired serious infections | OR 1.3, 95% CI (0.5-3.2) | [35] |
|  | Multi-site case-control study among children and adults with SARI or ILI | AF 85.6%, 95% CI (72.0-92.6%) | [36] |
|  | Nested case-control study among children 0-42-month-old with pneumonia | OR 1.12, 95% CI (0.67-1.88) | [37] |
|  | Multi-site case-control study among children and adults with a community-acquired pneumonia | AF 90%, 95% CI (80-95%)  OR 10.4, 95% CI (5.02-21.6) | [38] |
| Para-influenza virus  (PIV1-3) | Multi-site case-control study among children and adults with a community-acquired pneumonia | Children: OR 2.29, 95% CI (1.11-4.69)  Adults: p-value .09, OR NC | [38] |
|  | Multi-site case-control study among children and adults with SARI | Children:  PIV-1: OR 4.1, 95% CI (1.7-10.0)  PIV-2: OR 5.6, 95% CI (0.6-49.4)  PIV-3: OR 2.8, 95% CI (1.5-5.5)  Adults:  PIV-1: OR 1.7, 95% CI (0.3-10.3)  PIV-2: OR NC  PIV-3: OR 2.5, 95% CI (0.8-7.5) | [36] |
|  | Multi-site international case-control study among 1-59-month-old children with respiratory symptoms | PIV-1: OR 7.52, 95% CI (4.79-11.80)  PIV-2: OR 0.98, 95% CI (0.63-1.51)  PIV-3: OR 2.62, 95% CI (2.08-3.29) | [34] |
|  | Multi-site international case-control study among 0-59-day-old babies with community-acquired serious infections | PIV-1: OR 1.3, 95% CI (0.6-3.0)  PIV-2: OR 0.4, 95% CI (0.1-1.0)  PIV-3: OR 1.5, 95% CI (0.9-2.6) | [35] |
| Coronavirus  (HKU1; NL63; OC43; 229E) | Multi-site case-control study among children and adults with a community-acquired pneumonia | Children: aOR 3.17, 95% CI (1.44-6.99)  Adults: aOR 3.19, 95% CI (0.59-17.1) | [38] |
|  | Multi-site case-control study among children with an ARI | All coronavirus species:  aOR 0.79, 95% CI (0.53-1.17) | [39] |
|  | Prospective longitudinal study among children with an ARI | All coronavirus: asymptomatic 8% *versus* symptomatic 9%, p-value 0.38 | [40] |
|  | Multi-site international case-control study among 1-59-month-old children with respiratory symptoms | NL63: OR 0.77, 95% CI (0.58-1.03)  HKU1: OR 0.84, 95% CI (0.60-1.18)  229E: OR 0.76, 95% CI (0.48-1.22) | [34] |
| Rhinovirus  (RV) | Multi-site international case-control study among 1-59-month-old children with respiratory symptoms | OR 0.94, 95% CI (0.82-1.08) | [34] |
|  | Multi-site international case-control study among 0-59-day-old babies with community-acquired serious infections | OR 0.8, 95% CI (0.7-0.9) | [35] |
|  | Multi-site case-control study among children and adults with a community-acquired pneumonia | Children: OR 1.13, 95% CI (0.84-1.51)  Adults: OR 13.4, 95% CI (3.04-59.1) | [38] |
| *Bordetella pertussis* | Multi-site international case-control study among 1-59-month-old children with respiratory symptoms | OR 3.3, 95% CI (1.6-7.2) | [34] |
|  | Nested case-control study among 0-42-month-old children with pneumonia | OR 11.08, 95% CI (1.33-92.54) | [37] |

**Supplementary table 5.** Patient characteristics by organism detected in blood specimens in Chiang Rai, northern Thailand and Hlaing Tha Yar, Lower Myanmar, 2016-2017. Patients are presented by incremental C-reactive protein (CRP) concentrations.

| **Country** | **Methods** | **CRP** | **Age** | **Comorbidity** | **Symptom**  **(in days)** | **Tympanic** | **Clinical** | **Health worker** | **Antibiotic** |
| --- | --- | --- | --- | --- | --- | --- | --- | --- | --- |
| **Myanmar (n=50)** |  | **(mg/L)** | **(years)** |  | **onset (days)** | **temperature** | **presentation** | **diagnosis** | **prescription** |
| *Aerococcus* spp. | Singleplex PCR | 15 | 22 | Hepatitis B | 3 | 36.7 ˚C | Neurological & Respiratory | URTI | No |
| *Leptospira* spp. | Singleplex PCR | 8 | 8 | No | 2 | 37.0 ˚C | Undifferentiated | Acute viral infection | No |
|  | Singleplex PCR | 8 | 33 | Asthma | 4 | 37.6 ˚C | Undifferentiated | Acute viral infection | No |
|  | TAC | 62 | 15 | No | 2 | 39.2 ˚C | Respiratory | URTI | No |
|  | MAT | 63 | 29 | No | 3 | 38.6 ˚C | Respiratory & Digestive | URTI | Azithromycin |
|  | Singleplex PCR | 200 | 13 | No | 3 | 39.0 ˚C | Respiratory & Digestive | URTI | Amoxicillin |
|  | Singleplex PCR | 200 | 14 | No | 5 | 37.5 ˚C | Neurological & Digestive | - | No |
| *Klebsiella pneumoniae* | TAC | 8 | 6 | No | 2 | 38.4 ˚C | Respiratory | URTI | No |
|  | TAC | 10 | 11 | No | 3 | 38.4 ˚C | Respiratory | URTI | No |
|  | TAC | 10 | 56 | No | 4 | 37.0 ˚C | Neurological | - | Amoxicillin |
|  | TAC | 18 | 25 | HIV | 6 | 37.4 ˚C | Undifferentiated | HIV infection stage I | No |
|  | TAC | 28 | 37 | No | 3 | 37.3 ˚C | Respiratory | Acute viral infection | Amoxicillin |
|  | TAC | 44 | 7 | - | 1 | 37.7 ˚C | Digestive | Acute viral infection | No |
|  | TAC | 53 | 8 | No | 3 | 37.5 ˚C | Digestive | - | No |
|  | TAC | 98 | 7 | No | 1 | 37.5 ˚C | Respiratory | URTI | No |
| *Rickettsia* genus | IFA | 29 | 32 | No | 5 | 36.9 ˚C | Neurological & Digestive | Acute gastritis | No |
| *Salmonella* Paratyphi A | TAC | 26 | 32 | No | 3 | 36.6 ˚C | Digestive | Acute viral infection | No |
| *Streptococcus suis* | TAC | 16 | 12 | No | 4 | 37.9 ˚C | Respiratory | URTI | Amoxicillin |
|  | TAC | 200 | 27 | No | 7 | 37.2 ˚C | Respiratory | LRTI | Amoxicillin |
| *Streptococcus* spp. | Singleplex PCR | 12 | 28 | No | 2 | 37.1 ˚C | Respiratory & Digestive | URTI | Amoxicillin |
| Bocavirus | TAC | 97 | 43 | No | 5 | 38.8 ˚C | Respiratory | - | Amoxicillin |
| Dengue virus | Singleplex PCR | - | 2 | No | 1 | 38.3 ˚C | Digestive | Acute gastroenteritis | Ciprofloxacin |
|  | Singleplex PCR | - | 2 | No | 2 | 40.4 ˚C | Digestive | Chronic suppurative otitis media | Cloxacillin |
|  | Singleplex PCR | - | 4 | No | 1 | 39.5 ˚C | Digestive | Food poisoning | Ciprofloxacin |
|  | Singleplex PCR | - | 4 | No | 2 | 39.6 ˚C | Neurological & Digestive | Acute viral infection | No |
|  | Singleplex PCR | - | 4 | No | 4 | 38.6 ˚C | Neurological, Respiratory & Digestive | URTI | No |
|  | TAC | 8 | 9 | No | 4 | 37.5 ˚C | Undifferentiated | Acute viral infection | Amoxicillin |
|  | Singleplex PCR & TAC | 8 | 8 | No | 2 | 37.7 ˚C | Digestive | Acute gastroenteritis | No |
|  | Singleplex PCR & TAC | 8 | 13 | No | 1 | 39.9 ˚C | Respiratory | URTI | No |
|  | TAC | 8 | 13 | No | 1 | 38.4 ˚C | Neurological | URTI | No |
|  | Singleplex PCR & TAC | 8 | 16 | No | 1 | 38.8 ˚C | Respiratory | Acute viral infection | No |
|  | Singleplex PCR & TAC | 8 | 20 | No | 1 | 38.8 ˚C | Neurological & Respiratory | URTI | Amoxicillin |
|  | TAC | 9 | 6 | No | 5 | 37.1 ˚C | Neurological, Respiratory & Digestive | Acute viral infection | Cephalexin |
|  | Singleplex PCR & TAC | 10 | 7 | No | 5 | 37.5 ˚C | Undifferentiated | - | No |
|  | Singleplex PCR | 10 | 13 | No | 5 | 38.4 ˚C | Respiratory & Digestive | URTI | Azithromycin |
|  | Singleplex PCR & TAC | 10 | 6 | No | 2 | 38.6 ˚C | Respiratory | LRTI | Amoxicillin |
|  | TAC | 11 | 11 | No | 1 | 39.2 ˚C | Neurological | - | No |
|  | Singleplex PCR & TAC | 14 | 21 | No | 7 | 39.7 ˚C | Respiratory | Acute viral infection | No |
|  | Singleplex PCR & TAC | 15 | 8 | No | 2 | 37.6 ˚C | Digestive | Acute viral infection | No |
|  | Singleplex PCR & TAC | 15 | 12 | No | 1 | 39.0 ˚C | Respiratory & Digestive | Acute viral infection | No |
|  | Singleplex PCR & TAC | 17 | 18 | No | 3 | 39.1 ˚C | Digestive | Acute viral infection | No |
|  | Singleplex PCR & TAC | 20 | 10 | No | 5 | 38.7 ˚C | Undifferentiated | LRTI | Amoxicillin |
|  | Singleplex PCR & TAC | 31 | 7 | No | 1 | 37.6 ˚C | Neurological & Digestive | Head trauma | No |
|  | Singleplex PCR & TAC | 32 | 7 | No | 1 | 40.0 ˚C | Respiratory | LRTI | Amoxicillin |
|  | Singleplex PCR & TAC | 32 | 9 | No | 2 | 37.6 ˚C | Respiratory & Digestive | Acute viral infection | No |
|  | Singleplex PCR & TAC | 36 | 7 | No | 1 | 39.2 ˚C | Undifferentiated | Acute viral infection | No |
|  | Singleplex PCR & TAC | 43 | 5 | No | 2 | 39.6˚C | Undifferentiated | URTI | No |
|  | Singleplex PCR & TAC | 48 | 25 | No | 5 | 38.1 ˚C | Neurological | Non-specific fever | Azithromycin |
|  | Singleplex PCR & TAC | 105 | 8 | No | 1 | 40.2 ˚C | Neurological | - | No |
| Enterovirus | TAC | 8 | 6 | No | 3 | 37.6 ˚C | Respiratory & Digestive | URTI | Amoxicillin |

| **Thailand** | **Methods** | **CRP** | **Age** | **Comorbidity** | **Symptom onset**  **(in days)** | **Tympanic** | **Clinical** | **Health worker** | **Antibiotic** |
| --- | --- | --- | --- | --- | --- | --- | --- | --- | --- |
| **(n=29)** |  | **(mg/L)** | **(years)** |  | **onset (days)** | **temperature** | **presentation** | **diagnosis** | **prescription** |
| *Haemophilus influenzae* | TAC | 28 | 4 | No | 1 | 36.9 ˚C | Respiratory | Common cold | No |
| *Leptospira* spp. | MAT | 30 | ≥12 | No | 1 | 36.8 ˚C | Neurological & Respiratory | Acute pharyngitis | Amoxicillin |
|  | Singleplex PCR | 112 | 64 | Hypertension | 4 | 37.8 ˚C | Respiratory & Digestive | Common cold | No |
|  | Singleplex PCR | 158 | 8 | No | 1 | 36.9 ˚C | Neurological, Respiratory & Digestive | Common cold | No |
| *Rickettsia* genus | Singleplex PCR | 10 | 8 | No | 2 | 37.0 ˚C | Neurological, Respiratory & Digestive | Acute tonsillitis | Amoxicillin |
|  | Singleplex PCR | 28 | 7 | No | 1 | 37.9 ˚C | Respiratory & Digestive | Acute pharyngitis | Amoxicillin |
| *Salmonella* Paratyphi A | TAC | 8 | 11 | No | 3 | 36.7 ˚C | Neurological & Respiratory | Common cold | No |
|  | TAC | 18 | 10 | No | 3 | 36.6 ˚C | Respiratory | Acute pharyngitis | Amoxicillin |
| *Salmonella* Paratyphi A & *Rickettsia* genus | TAC | 8 | 11 | No | 1 | 39.6 ˚C | Respiratory | Acute pharyngitis | Amoxicillin |
| *Salmonella* spp. | TAC | 9 | 62 | Hypertension | 7 | 36.9 ˚C | Respiratory | Common cold | No |
|  | TAC | 14 | 20 | No | 2 | 36.7 ˚C | Neurological & Respiratory | Acute pharyngitis | No |
| *Streptococcus suis* | TAC | 8 | 5 | No | 1 | 37.8 ˚C | Respiratory | Common cold | No |
|  | TAC | 20 | 9 | No | 2 | 36.4 ˚C | Respiratory & Digestive | Common cold | No |
| Dengue virus | Singleplex PCR & TAC | 12 | 3 | No | 2 | 39.2 ˚C | Neurological, Respiratory & Digestive | Acute pharyngitis | Amoxicillin |
|  | Singleplex PCR & TAC | 23 | 9 | No | 2 | 38.6 ˚C | Neurological, Respiratory & Digestive | Fever of unknown cause | No |
| Enterovirus | TAC | 10 | 12 | No | 1 | 39.1 ˚C | Neurological & Respiratory | Acute tonsillitis | Amoxicillin |
|  | TAC | 12 | 8 | No | 1 | 38.5 ˚C | Respiratory | Acute pharyngitis | Amoxicillin |
|  | TAC | 13 | 4 | No | 3 | 37.4 ˚C | Respiratory | Common cold | No |
|  | TAC | 19 | 5 | No | 3 | 35.7 ˚C | Neurological & Respiratory | Common cold | No |
|  | TAC | 20 | 6 | No | 1 | 36.9 ˚C | Undifferentiated | Acute pharyngitis | Amoxicillin |
|  | TAC | 32 | 9 | No | 1 | 37.2 ˚C | Undifferentiated | Acute tonsillitis | Amoxicillin |
|  | TAC | 47 | 1 | No | 2 | 37.0 ˚C | Respiratory | Acute pharyngitis | Amoxicillin |
| Rhinovirus | TAC | 8 | 1 | No | 1 | 37.6 ˚C | Respiratory | Common cold | No |
|  | TAC | 8 | 2 | No | 1 | 37.0 ˚C | Respiratory | Common cold | No |
|  | TAC | 9 | 11 | No | 2 | 37.1 ˚C | Respiratory | Acute pharyngitis | Amoxicillin |
|  | TAC | 33 | 3 | No | 2 | 37.8 ˚C | Respiratory | Common cold | No |
| Rubella virus | TAC | 8 | 15 | No | 3 | 36.7 ˚C | Neurological, Respiratory & Digestive | Acute tonsillitis | Amoxicillin |
| Varicella-Zoster virus | TAC | 8 | 11 | No | 1 | 37.0 ˚C | Digestive | Common cold | No |
|  | TAC | 13 | 7 | No | 1 | 37.0 ˚C | Neurological | Varicella | Amoxicillin |

**Supplementary table 6.** Outcome characteristics among patients with a bacterial organism by antibiotic prescription in Chiang Rai, northern Thailand and Hlaing Tha Yar, Lower Myanmar, 2016-2017

| **Bacterial organism detected**  **(n=36)** | **No/No effective antibiotic prescribed (n=28) ^*^** | **Effective antibiotic prescribed (n=8)** | **p-value** |
| --- | --- | --- | --- |
| **Outcome characteristics** |  |  |  |
| Symptom resolution at day 5, n (%) | 16 (57.1) | 7 (87.5) | 0.170 |
| Symptom severity at day 5, median (IQR) | 1 (1-1) | 1 (1-1) | 1.000 |
| Documented fever at day 5, n (%) | 2 (7.1) | 0 (0) | 0.430 |
| Elevated CRP at day 5, n (%) | 1 (3.6) | 0 (0) | 0.583 |
| Symptom severity at day 14, median (IQR) | 1 (1-2) | - | - |
| Symptom resolution at day 14, n (%) | 27 (96.4) | 8 (100.0) | 0.747 |
| Symptom severity at day 14, median (IQR) | 1 (1-1) | - | - |
| Documented fever at day 14, n (%) | 0 (0) | 0 (0) | 1.000 |
| Occurrence of SAE, n (%) | 0 | 0 | 1.000 |
| Unscheduled visits, n (%) | 0 | 0 | 1.000 |

^*^No/No effective antibiotic indicates the presence of a bacteria and either absence of antibiotic prescription (n=24/36, 66.7%) or prescription of ineffective antibiotic (n=4/36, 11.1%). In our study, ineffective antibiotic prescription included the prescription of cotrimoxazole or metronidazole to *Leptospira* spp.; macrolide or tetracycline to *Streptococcus suis*; beta-lactam antibiotics to *Rickettsia* genus; amoxicillin, quinolones, or cephalosporin to *Bordetella pertussis*.

The prescription of antibiotics at the facility was considered between the enrolment at Day 0 until Day 14 of the follow-up

Severity was ranked from 1-4 with severity=1 being the less severe presentation.

CRP: C-reactive protein

Elevated CRP defined as ≥50 mg/L in children and ≥100 mg/L in adults

SAE: serious adverse event including hospitalisation or death within 14 days of enrolment

**References**

1. Valette M, Fanget R, Burfin G, Shedden D, Lina B. Evaluation of suitability of various novel swab devices for the molecular detection of Influenza A from surveillance samples in France. 2012.

2. Diaz MH, Waller JL, Napoliello RA, Islam MS, Wolff BJ, Burken DJ, et al. Optimization of Multiple Pathogen Detection Using the TaqMan Array Card: Application for a Population-Based Study of Neonatal Infection. PloS one. 2013;8(6):e66183. Epub 2013/06/28. doi: 10.1371/journal.pone.0066183. PubMed PMID: 23805203; PubMed Central PMCID: PMCPMC3689704.

3. Liu J, Gratz J, Amour C, Kibiki G, Becker S, Janaki L, et al. A laboratory-developed TaqMan Array Card for simultaneous detection of 19 enteropathogens. Journal of clinical microbiology. 2013;51(2):472-80.

4. Rachwal PA, Rose HL, Cox V, Lukaszewski RA, Murch AL, Weller SA. The potential of TaqMan Array Cards for detection of multiple biological agents by real-time PCR. PloS one. 2012;7(4):e35971.

5. Centers for Disease Control Prevention. Unexplained Respiratory Disease Outbreak working group activities-worldwide, March 2007-September 2011. MMWR Morbidity and mortality weekly report. 2012;61(26):480.

6. Kodani M, Yang G, Conklin LM, Travis TC, Whitney CG, Anderson LJ, et al. Application of TaqMan® low density arrays for simultaneous detection of multiple respiratory pathogens. 2011.

7. Ginzinger DGJEh. Gene quantification using real-time quantitative PCR: an emerging technology hits the mainstream. 2002;30(6):503-12.

8. Wolff BJ, Bramley AM, Thurman KA, Whitney CG, Whitaker B, Self WH, et al. Improved Detection of Respiratory Pathogens Using High-Quality Sputum with TaqMan Array Card Technology. 2016:JCM. 01805-16.

9. Mayxay M, Sengvilaipaseuth O, Chanthongthip A, Dubot-Pérès A, Rolain J-M, Parola P, et al. Causes of fever in rural Southern Laos. 2015;93(3):517-20.

10. Chheng K, Carter MJ, Emary K, Chanpheaktra N, Moore CE, Stoesser N, et al. A prospective study of the causes of febrile illness requiring hospitalization in children in Cambodia. 2013;8(4):e60634.

11. Southeast Asia Infectious Disease Clinical Research Network. Causes and outcomes of sepsis in southeast Asia: a multinational multicentre cross-sectional study. The Lancet Global health. 2017;5(2):e157-e67. Epub 2017/01/21. doi: 10.1016/s2214-109x(17)30007-4. PubMed PMID: 28104185; PubMed Central PMCID: PMCPMC5332551.

12. Lubell Y, Blacksell SD, Dunachie S, Tanganuchitcharnchai A, Althaus T, Watthanaworawit W, et al. Performance of C-reactive protein and procalcitonin to distinguish viral from bacterial and malarial causes of fever in Southeast Asia. BMC infectious diseases. 2015;15(1):511.

13. Mueller TC, Siv S, Khim N, Kim S, Fleischmann E, Ariey F, et al. Acute undifferentiated febrile illness in rural Cambodia: a 3-year prospective observational study. PloS one. 2014;9(4):e95868.

14. Wangrangsimakul T, Althaus T, Mukaka M, Kantipong P, Wuthiekanun V, Chierakul W, et al. Causes of acute undifferentiated fever and the utility of biomarkers in Chiangrai, northern Thailand. PLoS neglected tropical diseases. 2018;12(5):e0006477.

15. Liu J, Ochieng C, Wiersma S, Ströher U, Towner JS, Whitmer S, et al. Development of a TaqMan array card for acute-febrile-illness outbreak investigation and surveillance of emerging pathogens, including Ebola virus. Journal of clinical microbiology. 2016;54(1):49-58.

16. Pabbaraju K, Wong S, Gill K, Fonseca K, Tipples GA, Tellier R. Simultaneous detection of Zika, Chikungunya and Dengue viruses by a multiplex real-time RT-PCR assay. Journal of clinical virology : the official publication of the Pan American Society for Clinical Virology. 2016;83:66-71. Epub 2016/09/11. doi: 10.1016/j.jcv.2016.09.001. PubMed PMID: 27614319.

17. Smit PW, Elliott I, Peeling RW, Mabey D, Newton PN. An overview of the clinical use of filter paper in the diagnosis of tropical diseases. The American journal of tropical medicine and hygiene. 2014;90(2):195-210.

18. Jiang J, Chan TC, Temenak JJ, Dasch GA, Ching WM, Richards AL. Development of a quantitative real-time polymerase chain reaction assay specific for Orientia tsutsugamushi. The American journal of tropical medicine and hygiene. 2004;70(4):351-6. Epub 2004/04/22. PubMed PMID: 15100446.

19. Henry KM, Jiang J, Rozmajzl PJ, Azad AF, Macaluso KR, Richards AL. Development of quantitative real-time PCR assays to detect Rickettsia typhi and Rickettsia felis, the causative agents of murine typhus and flea-borne spotted fever. Molecular and cellular probes. 2007;21(1):17-23. Epub 2006/08/09. doi: 10.1016/j.mcp.2006.06.002. PubMed PMID: 16893625.

20. Jiang J, Blair PJ, Felices V, Moron C, Cespedes M, Anaya E, et al. Phylogenetic analysis of a novel molecular isolate of spotted fever group Rickettsiae from northern Peru: Candidatus Rickettsia andeanae. Annals of the New York Academy of Sciences. 2005;1063:337-42. Epub 2006/02/17. doi: 10.1196/annals.1355.054. PubMed PMID: 16481537.

21. Thaipadungpanit J, Chierakul W, Wuthiekanun V, Limmathurotsakul D, Amornchai P, Boonslip S, et al. Diagnostic accuracy of real-time PCR assays targeting 16S rRNA and lipL32 genes for human leptospirosis in Thailand: a case-control study. PloS one. 2011;6(1):e16236. Epub 2011/02/02. doi: 10.1371/journal.pone.0016236. PubMed PMID: 21283633; PubMed Central PMCID: PMCPMC3026019.

22. Cherkaoui A, Emonet S, Ceroni D, Candolfi B, Hibbs J, Francois P, et al. Development and validation of a modified broad-range 16S rDNA PCR for diagnostic purposes in clinical microbiology. Journal of microbiological methods. 2009;79(2):227-31. Epub 2009/09/29. doi: 10.1016/j.mimet.2009.09.014. PubMed PMID: 19782706.

23. Weisburg WG, Barns SM, Pelletier DA, Lane DJ. 16S ribosomal DNA amplification for phylogenetic study. Journal of bacteriology. 1991;173(2):697-703. Epub 1991/01/01. PubMed PMID: 1987160; PubMed Central PMCID: PMCPMC207061.

24. Faine S, Adler B, Bolin C, Perolat P. Leptospira and leptospirosis, Melbourne. Australia: MediSci. 1999:259.

25. Lim C, Paris DH, Blacksell SD, Laongnualpanich A, Kantipong P, Chierakul W, et al. How to determine the accuracy of an alternative diagnostic test when it is actually better than the reference tests: a re-evaluation of diagnostic tests for scrub typhus using Bayesian LCMs. PloS one. 2015;10(5):e0114930.

26. Lim C, Blacksell SD, Laongnualpanich A, Kantipong P, Day NP, Paris DH, et al. Optimal Cutoff Titers for Indirect Immunofluorescence Assay for Diagnosis of Scrub Typhus. Journal of clinical microbiology. 2015;53(11):3663-6. Epub 2015/09/12. doi: 10.1128/jcm.01680-15. PubMed PMID: 26354819; PubMed Central PMCID: PMCPMC4609688.

27. Paris DH, Dumler JS. State of the art of diagnosis of rickettsial diseases: the use of blood specimens for diagnosis of scrub typhus, spotted fever group rickettsiosis, and murine typhus. Current opinion in infectious diseases. 2016;29(5):433-9. Epub 2016/07/19. doi: 10.1097/qco.0000000000000298. PubMed PMID: 27429138; PubMed Central PMCID: PMCPMC5029442.

28. Desakorn V, Wuthiekanun V, Thanachartwet V, Sahassananda D, Chierakul W, Apiwattanaporn A, et al. Accuracy of a commercial IgM ELISA for the diagnosis of human leptospirosis in Thailand. The American journal of tropical medicine and hygiene. 2012;86(3):524-7. Epub 2012/03/10. doi: 10.4269/ajtmh.2012.11-0423. PubMed PMID: 22403329; PubMed Central PMCID: PMCPMC3284374.

29. Naigowit P LO, Yasang S, Biklang M, Warachit P. Development of a screening method for serodiagnosis of leptospirosis. International Medicine Journal of Thailand. 2001;17:182-7.

30. Jiang J, Chan TC, Temenak JJ, Dasch GA, Ching WM, Richards AL. Development of a quantitative real-time polymerase chain reaction assay specific for Orientia tsutsugamushi. The American journal of tropical medicine and hygiene. 2004;70(4):351-6. Epub 2004/04/22. PubMed PMID: 15100446.

31. Blacksell SD, Jenjaroen K, Phetsouvanh R, Wuthiekanun V, Day NP, Newton PN, et al. Accuracy of AccessBio Immunoglobulin M and Total Antibody Rapid Immunochromatographic Assays for the Diagnosis of Acute Scrub Typhus Infection. Clinical and vaccine immunology : CVI. 2010;17(2):263-6. Epub 2009/12/18. doi: 10.1128/CVI.00448-08. PubMed PMID: 20016046; PubMed Central PMCID: PMC2815529.

32. Henry KM, Jiang J, Rozmajzl PJ, Azad AF, Macaluso KR, Richards AL. Development of quantitative real-time PCR assays to detect Rickettsia typhi and Rickettsia felis, the causative agents of murine typhus and flea-borne spotted fever. Molecular and cellular probes. 2007;21(1):17-23. Epub 2006/08/09. doi: 10.1016/j.mcp.2006.06.002. PubMed PMID: 16893625.

33. Binder AM, Nichols Heitman K, Drexler NA. Diagnostic Methods Used to Classify Confirmed and Probable Cases of Spotted Fever Rickettsioses - United States, 2010-2015. MMWR Morb Mortal Wkly Rep. 2019;68(10):243-6. Epub 2019/03/15. doi: 10.15585/mmwr.mm6810a3. PubMed PMID: 30870409; PubMed Central PMCID: PMCPMC6421962 potential conflicts of interest. No potential conflicts of interest were disclosed.

34. O'Brien KL, Baggett HC, Brooks WA, Feikin DR, Hammitt LL, Higdon MM, et al. Causes of severe pneumonia requiring hospital admission in children without HIV infection from Africa and Asia: the PERCH multi-country case-control study. The Lancet. 2019.

35. Saha SK, Schrag SJ, El Arifeen S, Mullany LC, Islam MS, Shang N, et al. Causes and incidence of community-acquired serious infections among young children in south Asia (ANISA): an observational cohort study. 2018;392(10142):145-59.

36. Pretorius MA, Tempia S, Walaza S, Cohen AL, Moyes J, Variava E, et al. The role of influenza, RSV and other common respiratory viruses in severe acute respiratory infections and influenza-like illness in a population with a high HIV sero-prevalence, South Africa 2012-2015. Journal of clinical virology : the official publication of the Pan American Society for Clinical Virology. 2016;75:21-6. Epub 2016/01/08. doi: 10.1016/j.jcv.2015.12.004. PubMed PMID: 26741826; PubMed Central PMCID: PMCPMC5712432.

37. Zar HJ, Barnett W, Stadler A, Gardner-Lubbe S, Myer L, Nicol MP. Aetiology of childhood pneumonia in a well vaccinated South African birth cohort: a nested case-control study of the Drakenstein Child Health Study. The Lancet Respiratory medicine. 2016;4(6):463-72.

38. Self WH, Williams DJ, Zhu Y, Ampofo K, Pavia AT, Chappell JD, et al. Respiratory viral detection in children and adults: comparing asymptomatic controls and patients with community-acquired pneumonia. The Journal of infectious diseases. 2016;213(4):584-91.

39. Prill MM, Iwane MK, Edwards KM, Williams JV, Weinberg GA, Staat MA, et al. Human coronavirus in young children hospitalized for acute respiratory illness and asymptomatic controls. The Pediatric infectious disease journal. 2012;31(3):235-40. Epub 2011/11/19. doi: 10.1097/INF.0b013e31823e07fe. PubMed PMID: 22094637; PubMed Central PMCID: PMCPMC3288315.

40. van der Zalm MM, van Ewijk BE, Wilbrink B, Uiterwaal CS, Wolfs TF, van der Ent CK. Respiratory pathogens in children with and without respiratory symptoms. The Journal of pediatrics. 2009;154(3):396-400. e1.
